# ERJ Advances: interventional bronchoscopy

**DOI:** 10.1183/13993003.01946-2023

**Published:** 2024-07-11

**Authors:** Justin L. Garner, Pallav L. Shah, Felix Herth, Dirk-Jan Slebos

**Affiliations:** 1 Department of Lung Cancer and Interventional Bronchoscopy, Royal Brompton Hospital, London, UK; 2 National Heart and Lung Institute, Imperial College London, London, UK; 3 Department of Pneumology and Critical Care Medicine, Thoraxklinik and Translational Lung Research Center, Universität Heidelberg, Heidelberg, Germany; 4 Department of Pulmonary Diseases, University Medical Center Groningen, University of Groningen, Groningen, The Netherlands

## Abstract

The field of interventional bronchoscopy is rapidly growing, with the development of minimally invasive approaches and innovative devices to diagnose and treat a spectrum of respiratory diseases (figure 1), often as outpatient procedures, and supported by high quality collaborative research. This short review covers aspects related to COPD, peripheral pulmonary nodules, interstitial lung disease, and airway stenosis and malacia.

## Shareable abstract

**This ERJ Advances article summarises the latest developments in the rapidly advancing field of interventional bronchoscopy** https://bit.ly/44Qvgrm

## Introduction

**FIGURE 1:**
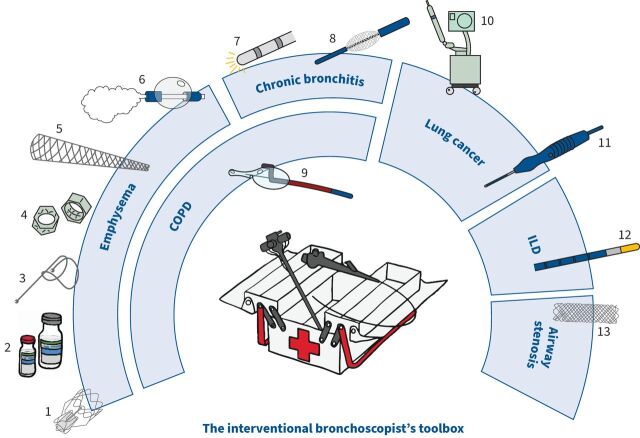
The interventional bronchoscopist's toolbox. Emphysema: 1) endobronchial valve, 2) Aeriseal, 3) endobronchial coil, 4) airway bypass stent, 5) implantable artificial bronchus and 6) bronchoscopic thermal vapour ablation. Chronic bronchitis: 7) metered radial cryospray and 8) rheoplasty. COPD with predominant airflow limitation: 9) targeted lung denervation. Peripheral pulmonary nodule: 10) ION robotic platform and 11) Creo Medical's bronchial microwave ablation catheter, MicroBlate Flex. Interstitial lung disease: 12) Erbe cryoprobe for transbronchial cryobiopsy. Airway stenosis: 13) biodegradable stent.

The field of interventional bronchoscopy is rapidly growing, with the development of minimally invasive approaches and innovative devices to diagnose and treat a spectrum of respiratory diseases (figure 1), often as outpatient procedures, and supported by high quality collaborative research. This short review covers aspects related to COPD, peripheral pulmonary nodules, interstitial lung disease, and airway stenosis and malacia.

## COPD

COPD is a complex inflammatory disorder of the small airways with a variety of manifestations of interest to the interventional bronchoscopist. The therapeutic approach to severe emphysema and hyperinflation continues to build on the proven reduction of excessive residual volume and restoration of normality to lung mechanics [1]. Current interests include the regeneration of a healthy functioning epithelium in patients with chronic bronchitis and the amelioration of frequent exacerbations using targeted lung denervation.

### Severe emphysema and hyperinflation

Approximately 1 in 100 patients with COPD are suitable for lung volume reduction. Evaluation is by a multidisciplinary team to ensure individuals have completed a programme of pulmonary rehabilitation, are taking optimal medical therapy, and are considered for all available approaches, including lung transplant.

Impairment of the passive elastic recoil mechanism, which in health maintains the patency of the small airways during expiration, results in an accumulation of trapped gases and a mechanical impediment to the ventilatory pump. Several devices with different modes of action have been developed to reduce hyperinflation.

Unidirectional valves induce atelectasis by occluding the segmental bronchi during inspiration, permitting evacuation of air and mucus in expiration. There are two main marketed devices: The Zephyr Endobronchial Valve (EBV) by Pulmonx Inc. (CA, USA), and the Spiration Valve System by Olympus (WA, USA). Absent interlobar collateral ventilation (CV), determined by surrogate quantification of fissure integrity on high resolution computed tomography and/or physiological measurement of lobar flow, is critical to success. The EBV, a duck-bill mechanism, has been the most studied and shown to improve lung function, exercise capacity, quality of life [2–4] and survival [5, 6] in selected individuals, and is a guideline-approved therapy; moreover, benefits are observed in homogeneous emphysema [7] and in lower lobe predominant disease [4]. Pneumothorax is the most frequent complication, occurring in up to 30% of recipients [3] and mandates a 72-h stay in hospital; management is usually by insertion of a 12-French thoracostomy tube [8]. A persistent air leak may require removal of one or more valves with staged re-implantation; reassuringly, outcomes are not adversely affected, provided complete lobar atelectasis is achieved [9]. Of those subjects screened in the TRANSFORM study, 16.5% were excluded owing to the presence of CV [3]. The CONVERT trial has been designed to broaden eligibility with instillations of Aeriseal sealant (Pulmonx Inc., CA, USA) into leaky fissures prior to EBV insertion: preliminary reports of up to an 83% “conversion” are most encouraging [10, 11].

Several alternative strategies to induce volume reduction independent of interlobar CV are the subject of research.

Endobronchial coils by PneumRx (CA, USA) are proposed to work by gathering the surrounding lung parenchyma and re-tensioning the airway network. They have shown promise, especially in patients with severe hyperinflation (residual volume >225% best predicted) and homogeneous emphysema deemed unsuitable for transplantation [12–16]; however, the technology has been withdrawn for financial reasons. A similar lung tensioning device system by Free Flow Medical (CA, USA) is currently under trial evaluation (NCT04520152). Another coil-shaped device from Lifetech Medical (Shenzhen, China) is being evaluated in a randomised controlled trial in China after finishing the first in-human study (NCT03685526).

Airway bypass, creating non-anatomical transbronchial fenestrations at the segmental level, supported by self-expanding stents, proved the concept. However, the benefits were short-lived despite paclitaxel elution and patency was not sustained [17]. The Pulmair (CA, USA) “Implantable Artificial Bronchus” instead stents the lobar segmental airways out to the 15th generation and counteracts expiratory airways collapse, facilitating gas emptying (US20180344445A1) [18], and is being prospectively evaluated in a multicentre trial (NCT05087641). An alternative embodiment by Apreo Health Inc. (CA, USA) employs an innovative geometric implant design to maintain luminal patency (US20220280279A1) and is under evaluation in a multicentre trial (NCT05854550).

Bronchoscopic thermal vapour ablation (BTVA) by Uptake Medical (WA, USA) offers a non-mechanical method of segmental volume reduction, preserving less diseased tissue and lessening the risk of pneumothorax, with promising results in individuals with heterogeneous upper-lobe predominant emphysema and hyperinflation [19, 20]. A post-market BTVA registry is in progress (NCT03318406), and another randomised controlled trial was recently approved in Germany (NCT05717192). Morair Medtech (WA, USA) have produced a similar system, endobronchial thermal liquid ablation, which uses heated normal saline (ACTRN12622001327774).

### Chronic bronchitis

Clinically defined by cough and sputum expectoration occurring on most days for at least three months of two consecutive years, chronic bronchitis is associated with frequent exacerbations and hospitalisations, accelerated lung function decline, poor quality of life, and reduced life expectancy [21]. A novel approach to reverse airways metaplasia and chronic mucus hypersecretion is selective cellular ablation, preserving a framework of extracellular structures, which is followed by healthy tissue regeneration – two such bronchoscopic epithelial resurfacing technologies, under trial evaluation, are described below.

The RejuvenAir system by CSA Medical (MA, USA) employs radial metered cryospray to flash freeze the epithelial lining at −196°C, inducing intracellular ice crystal formation, disrupting cellular structures but sparing the extracellular matrix. At 12 months, the treatment was shown to be safe, feasible, well-tolerated and associated with clinically meaningful improvements in cough, sputum production, breathlessness and quality of life [22]. A randomised sham-controlled study is under way to confirm the benefits and durability of this treatment in a larger population of patients (NCT03893370).

Bronchial rheoplasty by Galvanize Therapeutics Inc. (CA, USA) utilises pulsed electric fields to ablate the mucosal lining. Similarly encouraging benefits in patient-reported outcomes have been published [23] and a large, randomised sham-controlled trial is in progress (NCT04677465). A German–Austrian registry recently completed enrolment and will provide real world data (NCT04182841).

### Frequent exacerbations

Pharmacological blockade of the vagal innervation of the lungs results in bronchodilation, improved ciliary function, and reduced mucus secretion and exacerbation frequency [24]. Limitations to inhaled therapy include adherence and short duration of action. Targeted lung denervation, delivering circumferential radiofrequency ablation to the main bronchi in one treatment session, offers an alternative and more durable means of attenuating the overactive parasympathetic tone [25]. Employment of a radio-opaque gastro-oesophageal balloon serves to minimise gastric vagal plexus capture. The randomised double-blind sham-controlled trial, Airflow-2, showed clinically meaningful reductions in severe exacerbation frequency requiring hospitalisation [26]. A pivotal trial, Airflow-3, is designed to evaluate the safety and efficacy of targeted lung denervation in reducing moderate to severe exacerbations over 1 year [27].

## The peripheral pulmonary nodule

Lung cancer is the leading oncological cause of death worldwide [28]. Presentation is often advanced and prognosis consequently poor: 5-year survival is 25% for non-small cell lung carcinoma and 7% for small cell carcinoma [29]. The National Lung Cancer Screening Trial employing low-dose computed tomography (CT) demonstrated a risk reduction in mortality of 20% in individuals who were former smokers with a 30-pack year history [30].

### Diagnosis

Most incidental pulmonary nodules are found in the periphery of the lung and undergo surveillance as guided by predictive models [31]. A minority require interrogation and traditionally this is undertaken using transthoracic needle biopsy. The diagnostic yield is high (93%, 95% CI 90–96%) [32], offset by the risk of pneumothorax of up to 16%, with 6.6% requiring drainage [33].

Navigation bronchoscopy is an alternative approach embracing a range of techniques: virtual bronchoscopy, electromagnetic navigation, radial endobronchial ultrasound (REBUS), tomosynthesis, cone-beam computed tomography (CBCT), slimline scopes, robotic assistance, and combinations of these [34]. A meta-analysis of 126 studies comprising 16 077 patients with 16 389 lesions reported a pooled diagnostic yield of 69.4% (95% CI 0.67–0.71), with substantial variation among studies (40% to 97%) and significant between-study heterogeneity [35]. There was no difference in yield when comparing technologies; however, larger nodule size and the presence of a bronchus sign were associated with improved outcomes. A pneumothorax rate of 2.1% was quoted.

Robotic-assisted bronchoscopy is designed to optimise tool placement within a lesion: bespoke ventilation protocols [36] and real-time imaging feedback mitigate pre-procedural CT-to-body divergence [37]. The first prospective multicentre trial published a diagnostic yield of 74.1% (95% CI 61–84%) and pneumothorax rate of 3.7% [38]. A promising paradigm from the USA combines a shape-sensing robotic platform with REBUS and CBCT [39, 40] and is under trial evaluation in the UK (NCT05867953).

With the advent of nationwide lung cancer screening programmes, navigation bronchoscopy is likely to become a widespread complementary technology with multimodal sampling approaches adopted, facilitated by advanced imaging adjuncts and rapid on-site evaluation (human or artificial intelligence driven) [41], to maximise diagnostic yields and reduce procedural times.

### Therapy

Surgical resection is the preferred treatment modality for peripheral early-stage non-small cell lung carcinoma [42–44]. For those individuals who decline surgery or in whom the risk is prohibitive, stereotactic body radiation therapy (SBRT) [45, 46] and percutaneous ablative techniques [47] are established alternatives.

An alternative approach under trial evaluation is bronchoscopy-delivered transbronchial ablation employing thermal (microwave: NCT05299606, NCT05281237, NCT05786625; cryotherapy: NCT04049474) and non-thermal (brachytherapy and photodynamic therapy) energy sources. The use of localised ablation to release tumour antigen into the circulation and potentiate the effects of immunotherapy is currently being evaluated (NCT05053802, NCT04793815). Expanding indications may include individuals with unresectable local recurrence after surgery or SBRT and biopsy-proven synchronous lesions. It remains to be seen whether the impact of bronchoscopy ablation on local recurrence rates and survival compares favourably with current standard of care therapies.

## Interstitial lung disease

The diffuse parenchymal lung diseases encompass more than 200 conditions characterised by inflammation or fibrosis of the alveolar-capillary compartment. There is significant overlap in presentation but differences in treatment response and prognosis make diagnosis challenging; multidisciplinary discussion integrating clinical, radiological, serological and histological data is fundamental to this process [48]. Surgical lung biopsy is regarded as the gold standard modality for tissue acquisition, but is associated with complications including persistent air leak in up to 5% [49], exacerbation of the underlying disease process in 7%, major bleeding in 2.2% [50], and 30-day mortality in 2.4% (similar to lobectomy for lung cancer) [51]. Transbronchial lung cryobiopsy offers a minimally invasive means of procuring tissue with a comparable diagnostic accuracy [52], and substantially lower morbidity and mortality [53]. The architectural preservation of the sample not subject to the crush artefacts seen with mechanical forceps transbronchial biopsies permits a more detailed histological characterisation with similar prognostic value to surgical lung biopsy [54]. The technique is now incorporated into society guidelines [55]. Procedural modifications employing advanced imaging adjuncts such as radial endobronchial ultrasound [56] and cone-beam computed tomography [57] and the addition of a genomic classifier [58] have been proposed to improve diagnostic yield.

## Airway stenosis and malacia

### Three-dimensional stent printing

The treatment of benign and malignant airway stenoses frequently poses a challenge owing to complex anatomy with individual variation. Commercially available stents not infrequently suffer migration and granulation tissue reaction, leading to luminal occlusion. A number of software platforms exist that can segment anatomical structures including the airway tree to a high resolution. Three-dimensional modelling and printing circumvents the “one-glove-fits-all” paradigm and permits the manufacture of patient-specific devices, with promising preliminary results published [59, 60]. The use of novel biocompatible materials with antimicrobial properties [61] will minimise the risk of endoluminal tissue encroachment and infection. The process is seemingly applicable to any form of bronchial implant, which holds great potential in the pursuit of personalised medicine.

### Biodegradable stents

Problems common to silicone and to metal (nitinol and medical grade stainless steel) stents are an aggressive granulation response and biofilm formation, both degrading their function and necessitating further bronchoscopic procedures to clean or replace them. Stenotic and malacic airway pathologies are especially prevalent after lung transplantation. For this indication, and perhaps for other benign and longer lasting malignant problems, biodegradable ELLA stents (ELLA-CS, Hradec Králové, Czech Republic) have been suggested [62, 63].

## Conclusion

This short review summarises the latest bronchoscopic innovations and advances in response to the demand for minimally invasive management of a broadening spectrum of problems. Evolution of the speciality will depend on randomised sham-controlled double-blind trials and head-to-head comparisons of technologies with capture of important end-points (for example, requirement for repeat procedures, impact on quality of life) to determine the most appropriate modalities and management algorithms for our patients. The future is promising.

## Shareable PDF

10.1183/13993003.01946-2023.Shareable1 This one-page PDF can be shared freely online.
Shareable PDF ERJ-01946-2023.Shareable
