# Supplementary material for: ERJ Advances: interventional bronchoscopy
Source: Eur Respir J. 2024 Jul 11;64(1):2301946. doi: 10.1183/13993003.01946-2023 (PMC11540446; doi:10.1183/13993003.01946-2023)
Supplement: Supplementary file 1 [file ERJ-01946-2023.Shareable.pdf]

EUROPEAN RESPIRATORY JOURNAL
EDITORIAL
J.L. GARNER ET AL.

SHAREABLE PDF

ERJ Advances: interventional bronchoscopy

Justin L. Garner [1,2], Pallav L. Shah [1,2], Felix Herth [3] and Dirk-Jan Slebos [4]

[1]Department of Lung Cancer and Interventional Bronchoscopy, Royal Brompton Hospital, London, UK. [2]National Heart and Lung Institute, Imperial College London, London, UK. [3]Department of Pneumology and Critical Care Medicine, Thoraxklinik and Translational Lung Research Center, Universität Heidelberg, Heidelberg, Germany. [4]Department of Pulmonary Diseases, University Medical Center Groningen, University of Groningen, Groningen, The Netherlands.

Corresponding author: Justin L. Garner (j.garner@rbht.nhs.uk)

Check for updates

Shareable abstract (@ERSpublications)
This ERJ Advances article summarises the latest developments in the rapidly advancing field of interventional bronchoscopy https://bit.ly/44Qvgrm

Cite this article as: Garner JL, Shah PL, Herth F, *et al*. ERJ Advances: interventional bronchoscopy. *Eur Respir J* 2024; 64: 2301946 [DOI: 10.1183/13993003.01946-2023].

This extracted version can be shared freely online.

Copyright ©The authors 2024. For reproduction rights and permissions contact permissions@ersnet.org

Received: 2 Nov 2023
Accepted: 14 May 2024

The field of interventional bronchoscopy is rapidly growing, with the development of minimally invasive approaches and innovative devices to diagnose and treat a spectrum of respiratory diseases (figure 1), often as outpatient procedures, and supported by high quality collaborative research. This short review covers aspects related to COPD, peripheral pulmonary nodules, interstitial lung disease, and airway stenosis and malacia.
